# Diversity of *Xenorhabdus* and *Photorhabdus* spp. and Their Symbiotic Entomopathogenic Nematodes from Thailand

**DOI:** 10.1371/journal.pone.0043835

**Published:** 2012-09-12

**Authors:** Aunchalee Thanwisai, Sarunporn Tandhavanant, Natnaree Saiprom, Nick R. Waterfield, Phan Ke Long, Helge B. Bode, Sharon J. Peacock, Narisara Chantratita

**Affiliations:** 1 Department of Microbiology and Immunology, Faculty of Tropical Medicine, Mahidol University, Bangkok, Thailand; 2 Mahidol-Oxford Tropical Medicine Research Unit, Faculty of Tropical Medicine, Mahidol University, Bangkok, Thailand; 3 Department of Biology and Biochemistry, University of Bath, Bath, United Kingdom; 4 Vietnam National Museum of Nature, Vietnam Academy of Science and Technology, Caugiay, Hanoi, Vietnam; 5 Molecular Biotechnology, Institute for Molecular Biosciences, Goethe University Frankfurt, Frankfurt, Germany; 6 Department of Medicine, University of Cambridge, Addenbrooke's Hospital, Cambridge, United Kingdom; Universidad Pública de Navarra, Spain

## Abstract

*Xenorhabdus* and *Photorhabdus* spp. are bacterial symbionts of entomopathogenic nematodes (EPNs). In this study, we isolated and characterized *Xenorhabdus* and *Photorhabdus* spp. from across Thailand together with their associated nematode symbionts, and characterized their phylogenetic diversity. EPNs were isolated from soil samples using a *Galleria*-baiting technique. Bacteria from EPNs were cultured and genotyped based on *recA* sequence. The nematodes were identified based on sequences of 28S rDNA and internal transcribed spacer regions. A total of 795 soil samples were collected from 159 sites in 13 provinces across Thailand. A total of 126 EPNs isolated from samples taken from 10 provinces were positive for *Xenorhabdus* (n = 69) or *Photorhabdus* spp. (n = 57). Phylogenetic analysis separated the 69 *Xenorhabdus* isolates into 4 groups. Groups 1, 2 and 3 consisting of 52, 13 and 1 isolates related to *X. stockiae*, and group 4 consisting of 3 isolates related to *X. miraniensis*. The EPN host for isolates related to *X. stockiae* was *S. websteri*, and for *X. miraniensis* was *S. khoisanae*. The *Photorhabdus* species were identified as *P. luminescens* (n = 56) and *P. asymbiotica* (n = 1). Phylogenenic analysis divided *P. luminescens* into five groups. Groups 1 and 2 consisted of 45 and 8 isolates defined as subspecies *hainanensis* and *akhurstii*, respectively. One isolate was related to *hainanensis* and *akhurstii*, two isolates were related to *laumondii*, and one isolate was the pathogenic species *P. asymbiotica* subsp. *australis*. *H. indica* was the major EPN host for *Photorhabdus*. This study reveals the genetic diversity of *Xenorhabdus* and *Photorhabdus* spp. and describes new associations between EPNs and their bacterial symbionts in Thailand.

## Introduction

*Xenorhabdus* and *Photorhabdus* spp. are bacterial symbionts of entomopathogenic nematodes (EPNs) belonging to the genera *Steinernema* and *Heterorhabditis*, respectively. These Gram-negative bacilli inhabit the intestine of infective juvenile stage EPNs present in soil. EPNs infect the larval stage of a diverse range of insects and release their bacterial cargo, which multiply and secrete proteins and secondary metabolites that are lethal to the insect larva and suppress the growth of other competing environmental bacteria, fungi, nematodes and protists. The dead insect cadaver provides a nutrient source for completion of the nematode lifecycle. When nutrients are depleted, the bacteria re-associate with the infective juvenile stage EPNs, which then disperse into the environment to search for a new insect host [1].

Sixty-one species of *Steinernema* and 24 species of *Heterorhabditis* nematodes have been identified to date [2], [3], [4]. A total of 21 *Xenorhabdus* spp. have been described in association with *Steinernema*, and 3 *Photorhabdus* spp. have been associated with *Heterorhabditis* (*P. luminescens*, *P. temperata* and the human pathogen *P. asymbiotica*) [5], [6]. The common bacterial-EPN associations are *P. luminescens* with *H. bacteriophora*, *H. indica* and *H. georgiana*; *P. temperata* with *H. bacteriophora*, *H. megidis*, *H. downesi*, *H. georgiana*, *H. marelatus* and *H. zealandica*; and *P. aymbiotica* with *H. gerradi* [6], [7], [8]. *Xenorhabdus* spp. are associated with a greater range of *Steinernema* hosts [6], [7]. EPNs are distributed worldwide, with distinctive species and clusters in different geographical regions. Nevertheless there is limited information on EPNs and their symbiotic bacteria in many countries, including Thailand. A study based on environmental sampling in central Thailand (Lohmsak, Phetchabun) reported the presence of *S. siamkayai* in association with the bacterial species *X*. *stockiae* [6], [7], and a second study reported *H. indica* in association with *P. luminescens* in Khon Kaen and Krabi in the northeast and southern Thailand, respectively [8]. These authors also identified a potentially new *Heterorhabditis* species (MP68) from Kanchanaburi in Western Thailand associated with *P. luminescens* [8]. The objectives of this study were to isolate and identify EPNs and their associated *Xenorhabdus* and *Photorhabdus* spp. from across Thailand and describe their phylogeography. We also defined the relationship between the bacteria and EPN species and defined the association of EPNs recovery and various soil parameters.

## Materials and Methods

### Collection of soil samples

A total of 795 soil samples from 159 sites were collected from 13 provinces in Thailand between 4 July 2009 and 22 October 2009 (the rainy season). Samples were taken from random areas of natural grassland, roadside verges, woodland, and the banks of ponds and rivers. No specific permits were required for the described field studies. For each site, 5 soil samples were randomly taken in an area of approximately 100 m^2^ at a depth of 10–20 cm using a hand shovel. Approximately 500 g of each soil sample was placed into a plastic bag. The longitude, latitude and altitude of each sampling site were recorded using GPSMAP 60CSx (Garmin, Taiwan). The temperature, pH and moisture of each sample were recorded using a Soil PH & Moisture Tester (Model: DM-15, Takemura electric works, Ltd, Japan). Soil samples were maintained at 25–32°C (ambient temperature) during transportation to our laboratory in Bangkok.

### Isolation of entomopathogenic nematodes (EPN)

EPNs were recovered from soil samples using an established insect (*Galleria mellonella*) baiting technique, as described previously [9]. In brief, each soil sample was placed into a plastic box and 5 last instar *G. mellonella* larvae were placed on the soil surface. The box lid was put in place, the box inverted and stored at 25°C for 5 days. Dead *G. mellonella* were collected and each *G. mellonella* cadaver placed into a White trap [10]. This was maintained in the dark at 20–25°C to allow for the emergence of infective juvenile nematodes (IJs). Soil samples associated with death of *G. mellonella* were re-baited three times using fresh larvae. Emergent nematodes were collected and pooled for a given soil sample and used to infect new *G. mellonella* larvae to confirm entomopathogenicity and to amplify the number of nematodes. This was performed by adding 500 µl containing approximately 100 EPNs onto a sterile petri dish. Two insect larvae were placed in the dish, which was sealed with parafilm and incubated in the dark at 25°C. The insects were observed daily, dead *G. mellonella* collected and IJs harvested as above. Nematodes were maintained at 13°C in distilled water prior to molecular characterisation.

### Isolation and identification of *Xenorhabdus* and *Photorhabdus* spp. from nematodes

Dead insects were cleaned by immersing in 75% ethanol prior to dissecting on a sterile petri dish. *Xenorhabdus* and *Photorhabdus* spp. were obtained by streaking 1 µl of haemolymph onto nutrient bromothymol blue agar (NBTA) [11]. Plates were sealed with parafilm and incubated at ambient temperature (25°C) for 4 days. In an initial pilot study of the first 35 soil samples, colonies of presumptive *Xenorhabdus* and *Photorhabdus* spp. on NBTA were selected based on colony morphology and the catalase test [11]. Twelve different colony types were identified which were further characterized using PCR and sequencing of a region of the 16S rRNA gene, as described previously [12], [13]. This demonstrated that 3 specific colony types were *Xenorhabdus* spp. and 4 specific colony types were *Photorhabdus* spp. Sequence analysis of a region of the 16S rRNA gene confirmed the presence of a region at position 208–211 (*E. coli* numbering) that distinguished between the two species (*Xenorhabdus* for TTCG and TGAAAG in *Photorhabdus*), as described previously [13]. Colony morphology was used thereafter to select colonies for further analysis. *Xenorhabdus* was characterized based on a dark blue or dark red colony colour with a convex or umbonated surface and swarming on NTBA after 3 to 4 days at room temperature (25°C), and catalase test negative. *Photorhabdus* was characterized based on a light or dark green colony colour with a convex or umbonated surface on NTBA after 3 to 4 days at room temperature (25°C), and catalase test positive.

Species identification and phylogenetic analysis of all bacterial isolates were subsequently performed on the basis of *recA* sequence. Total genomic DNA was extracted from a 3 ml LB overnight culture of *Xenorhabdus* or *Photorhabdus* spp. using a Genomic DNA Mini Kit (Geneaid Biotech Ltd., Taiwan). A 890 bp region of the *recA* gene was amplified by PCR using the following primers: recA_F (5′-GCTATTGATGAAAATAAACA-3′) and recA_R (5′- RATTTTRTCWCCRTTRTAGCT-3′) [6]. PCR was performed in a total volume of 50 ìl containing 3.5 mM MgCl_2_, 1 mM of each dNTP, 0.5 ìM of each primer, 0.05 unit of Taq DNA polymerase (Promega, USA), and 2.5 ìl of DNA in 1× reaction buffer using a PTC-200 Peltier Thermal Cycler (MJ research INC., Watertown, Massachusetts, USA). PCR cycling parameters were an initial step of 94°C for 5 min, followed by 30 cycles of 94°C for 1 min, 50°C for 1 min and 72°C for 2 min and a final extension of 72°C for 7 min. PCR products were visualized on ethidium bromide stained agarose-gel electrophoresis and purified using Gel/PCR DNA Fragment Extraction Kit (Geneaid Biotech Ltd., Taiwan). Purified PCR products were sequenced by Macrogen Inc. (Korea). Sequences have been deposited in Genbank under accession numbers JQ973956 to JQ974024 for *Xenorhabdus* spp., and JQ973899 to JQ973955 for *Photorhabdus* spp. Species identification was performed using a BLASTN search of *recA* against the NCBI nucleotide database (http://www.ncbi.nlm.nih.gov/blast/Blast.cgi), and the match with the highest similarity score selected. Multiple nucleotide sequences representing all of the known species and subspecies of *Photorhabdus* and *Xenorhabdus* spp. were downloaded from the NCBI database, aligned with sequences from the study isolates, and trimmed to a 646 bp region using Clustal W [14] using MEGA software version 5.05 [15]. Maximum likelihood trees were reconstructed using Nearest-Neighbor-Interchange (NNI) and Tamura-Nei model [16] using MEGA software version 5.05 [15]. Bootstrap analysis was carried out with 1,000 datasets.

### Molecular characterisation of entomopathogenic nematodes

Genomic DNA was extracted from approximately 50 infective juveniles for each sample, as described previously [17]. Molecular identification of *Steinernema* and *Heterorhabditis* spp. was performed by PCR amplification and sequencing of a region of the 28S rDNA gene and two ITS regions of the rDNA gene, respectively. The primers and methods used were as described previously [17], [18], with the exception that cycling conditions were modified to eradicate non-specific bands. These were as follows: 28S rDNA gene; 95°C for 5 min, 35 cycles of 94°C for 1 min, 55°C for 30 sec and 72°C for 45 sec, and a final extension at 72°C for 7 min; ITS region of the rDNA; 95°C for 5 min, 35 cycles of 94°C for 1 min, 50°C for 30 sec and 72°C for 1 min, and final extension at 72°C for 7 min. Amplified products were visualized on ethidium bromide stained agarose-gel electrophoresis and purified using a Gel/PCR DNA Fragments Extraction Kit (Geneaid Biotech Ltd., Taiwan). Sequencing was performed by Macrogen Inc. (Korea) and edited using SeqManII software (DNASTAR Inc., Wisconsin, USA). Species identification was performed using a BLASTN search against a nucleotide database (http://www.ncbi.nlm.nih.gov/blast/Blast.cgi).

### Data analysis

Statistical analysis was performed using STATA version 11 (Stata Corp, College Station, Tx, USA). The value of soil pH, temperature and moisture were compared between EPN positive and negative groups. These parameters were not normally distributed and were analysed using the Mann-Whitney test. Results were expressed as the median and interquartile range (IQR).

## Results

### Isolation of entomopathogenic nematodes and their symbiotic bacteria

**Figure 1:**
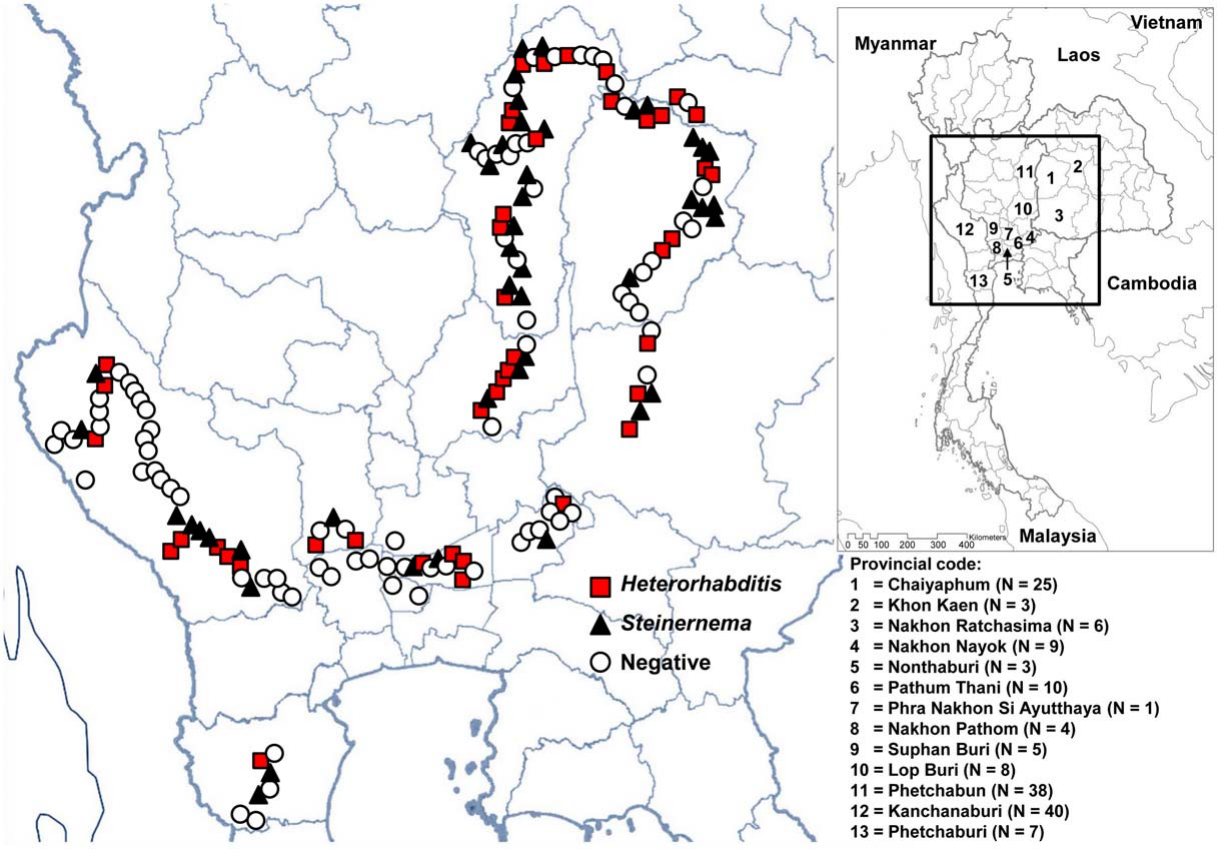
Geographical distribution of sample collection from 159 sites in Thailand and the distribution of sampling sites that were positive or negative for *Steinernema* and *Heterorhabditis*.

A total of 795 soil samples were collected from 159 sampling sites in 13 provinces (Table 1). EPNs positive for *Photorhabdus* or *Xenorhabdus* spp. were isolated from 88 sites located in the northeast (24/34 sites tested, 71%), central region (45/78, 58%), or the west (19/47, 40%) (Figure 1). A total of 126 EPNs were isolated, of which 69 were positive for *Xenorhabdus* spp. and 57 were positive for *Photorhabdus* spp. (Table 1). EPNs positive for *Photorhabdus* or *Xenorhabdus* spp. were most often isolated in only one out of five soil samples taken at a specific site (29/45 (64%) sites positive for *Xenorhabdus* spp. and 31/43 (72%) sites positive for *Photorhabdus* spp.). Two, 3, 4 or 5 samples were positive for *Xenorhabdus* spp. in 10, 4, 2 and 0 sites, respectively, and positive for *Photorhabdus* spp. in 10, 2, 0 or 0 sites, respectively. EPNs carrying *Xenorhabdus* spp. and EPNs carrying *Photorhabdus* spp. co-existed in same site for 10 sites, but no EPNs carried both species.

**Table 1:** Isolation of entomopathogenic nematodes and associated *Xenorhabdus* or *Photorhabdus* spp. from soil in Thailand.

Region	Province	Total sites	No. of sampling sites positive (%)			Total soil samples	No. of soil samples positive (%)		
			EPNs with *Xenorhabdus*	EPNs with *Photorhabdus*	All EPNs		EPNs with *Xenorhabdus*	EPNs with *Photorhabdus*	All EPNs
Northeast	Chaiyaphum	25	10	7	17	125	15	11	26
	Khon Kaen	3	0	2	2	15	0	2	2
	Nakhon Ratchasima	6	2	3	5	30	5	6	11
	SUBTOTAL	34	12 (35.3%)	12 (35.3%)	24 (70.6%)	170	20 (11.8%)	19 (11.2%)	39 (22.9%)
Central	Nakhon Nayok	9	1	1	2	45	1	2	3
	Nonthaburi	3	0	0	0	15	0	0	0
	Pathum Thani	10	2	4	6	50	2	4	6
	Phra Nakhon Si Ayutthaya	1	0	0	0	5	0	0	0
	Nakhon Pathom	4	0	0	0	20	0	0	0
	Suphan Buri	5	1	2	3	25	1	2	3
	Lop Buri	8	3	5	8	40	3	6	9
	Phetchabun	38	16	10	26	190	25	13	38
	SUBTOTAL	78	23 (29.5%)	22 (28.2%)	45 (57.7%)	390	32 (8.2%)	27 (6.9%)	59 (15.1%)
West	Kanchanaburi	40	8	8	16	200	12	9	21
	Phetchaburi	7	2	1	3	35	5	2	7
	SUBTOTAL	47	10 (21.3%)	9 (19.1%)	19 (40.4%)	235	17 (7.2%)	11 (4.7%)	28 (11.9%)
	TOTAL	159	45 (28.3%)	43 (27.0%)	88 (55.3%)	795	69 (8.7%)	57 (7.2%)	126 (15.8%)

### The effect of soil parameters on EPN isolation

The soil types for samples which yielded EPNs isolates were loamy (71%), sandy loam (17%), clay (9%) or sandy (3%), which was comparable to the distribution of soil type for samples which did not yield EPNs, which was loamy (66%), sandy loam (12%), clay (20%), and sandy (2%). The pH, temperature and moisture were recorded for 760/795 soil samples (119 EPN-positive and 641 EPN-negative). Soil pH ranged between 3.2 and 7.5 (median 6.3, interquartile range [IQR] 6.0–6.6). The pH of EPN-positive and EPN-negative soil was not significantly different ((range 3.2 to 6.9, median 6.4, IQR = 6.0–6.6) versus (range 3.2 to 7.5, median 6.3, IQR 5.9–6.6), respectively, *P* = 0.9). Soil temperature ranged between 23.0 and 37.0°C (median 28.5°C, IQR 27–30°C). The temperature of EPN-positive and EPN-negative soil was not significantly different ((range 24–32°C, median 28°C, IQR 27–29°C) versus (range 23–37°C, median 28.5°C, IQR 27–30°C), respectively, *P* = 0.08). Soil moisture ranged from 0 to 8.0% (median 4.0%, IQR 2.0–6.3%). The moisture of EPN-positive and EPN-negative soil was not significantly different (range 0.2 to 8%, median 3.5%, IQR 2.0–5.5%) versus (range 0 to 8%, median 4.0%, IQR 2.0–6.5%), respectively (*P* = 0.3).

### Identification and phylogenetic analysis of *Xenorhabdus* isolates

**Figure 2:**
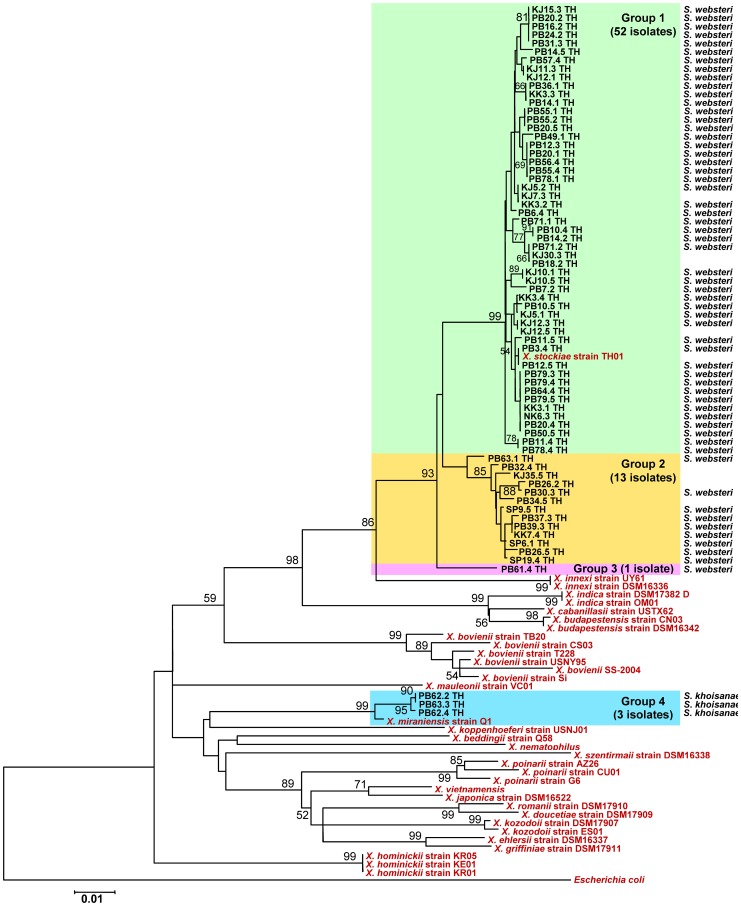
Maximum likelihood tree based on a 646 bp region of *recA* for 69 *Xenorhabdus* isolates from Thailand (codes ending with TH), together with *Xenorhabdus* sequences downloaded from the GenBank database (shown in red). Bootstrap values are based on 1,000 replicates. Numbers shown above branches are bootstrap percentages for clades supported above the 50% level. The bar indicates 1% sequence divergence. The EPN species from which they were isolated are shown.

The 69 *Xenorhabdus* isolates were identified as *X. stockiae* (n = 52), closely related to *X. stockiae* (n = 14), and closely related to *X. miraniensis* (n = 3). These findings were replicated in a phylogenetic analysis. A maximum likelihood tree reconstructed using the 69 *Xenorhabdus* sequences together with sequences downloaded from GenBank is shown in Figure 2. The Thai isolates fell into four distinct groups. Group 1 included 52 study isolates and a sequence from the NCBI database derived from *X. stockiae*. Group 2 included 13 study isolates but no reference sequence, and group 3 included one study isolate that was most closely related to group 2. The fourth cluster contained the remaining 3 Thai isolates, which were most closely related to *X. miraniensis*. Geographical mapping of the largest phylogenetic group containing the 52 *X. stockiae* isolates demonstrated that these were recovered from diverse geographical locations including 7 provinces (Table 2), as were the 13 isolates belonging to group 2.

**Table 2:** Geographical distribution of *Xenorhabdus* and *Photorhabdus* spp. isolated in Thailand.

Region	Province	Total Sampling sites	Total samples	Number of *Xenorhabdus* isolates				Number of *Photorhabdus* isolates				
				Group 1	Group 2	Group 3	Group 4	Group 1	Group 2	Group 3	Group 4	Group 5
				*X. stockiae*	Related to *X. stockiae*	Related to *X. stockiae*	Related to *X. miraniensis*	*P. luminescens*				*P.asymbiotica*
								subsp. *hainanensis*	subsp. *akhurstii*	Related to *hainanensis* & *akhurstii*	Related to *laumondii*	subsp. *australis*
Northeast	Chaiyaphum	25	125	10	1	1	3	6	0	0	2	1
	Khon Kaen	3	15	0	0	0	0	2	0	0	0	0
	Nakhon Ratchasima	6	30	5	0	0	0	6	0	0	0	0
Central	Nakhon Nayok	9	45	1	0	0	0	0	2	0	0	0
	Nonthaburi	3	15	0	0	0	0	0	0	0	0	0
	Pathum Thani	10	50	0	2	0	0	4	0	0	0	0
	Phra Nakhon Si Ayutthaya	1	5	0	0	0	0	0	0	0	0	0
	Nakhon Pathom	4	20	0	0	0	0	0	0	0	0	0
	Suphan Buri	5	25	0	1	0	0	1	1	0	0	0
	Lop Buri	8	40	3	0	0	0	6	0	0	0	0
	Phetchabun	38	190	18	7	0	0	11	3	1	0	0
West	Kanchanaburi	40	200	11	1	0	0	7	2	0	0	0
	Phetchaburi	7	35	4	1	0	0	2	0	0	0	0
TOTAL	13 provinces	159	795	52	13	1	3	45	8	1	2	1

### Identification and phylogenetic analysis of *Photorhabdus* isolates

**Figure 3:**
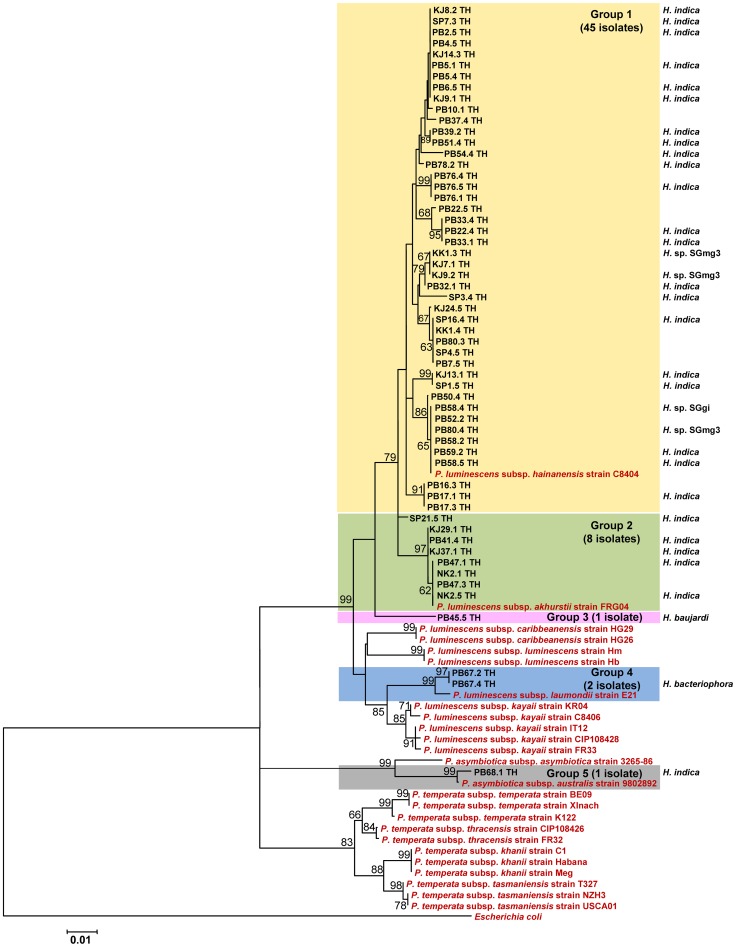
Maximum likelihood tree based on a 646 bp region of *recA* for 57 *Photorhabdus* isolates from Thailand (codes ending with TH), together with *Photorhabdus* sequences downloaded from GenBank (shown in red). **Bootstrap values are based on is 1,000 replicates.** Numbers shown above branches are bootstrap percentages for clades supported above the 50% level. The bar indicates 1% sequence divergence. The EPN species from which they were isolated are shown.

The 57 *Photorhabdus* isolates were identified as *P. luminescens* (n = 56) and the human pathogenic species *P. asymbiotica* (n = 1). The *P. luminescens* isolates were sub-speciated as subsp. *hainanensis* (n = 45), *P. luminescens* subsp. *akhurstii* (n = 8), a sub-species most closely related to subsp. *laumondii* (n = 2), or a subspecies related to *hainanensis* and *akhurstii* but no reference identified in the database (n = 1). The isolate of *P. asymbiotica* was identified as *P. asymbiotica* subsp. *australis*. These findings were replicated in a phylogenetic analysis. A maximum likelihood tree reconstructed using the 57 *Photorhabdus* sequences together with sequences downloaded from GenBank are shown in Figure 3. A total of 56 sequences clustered with sequences from known *P. luminescens* isolates, and one Thai isolate clustered with *P. asymbiotica* and was most closely related to *P. asymbiotica* subsp. *australis*. The sequences from the 56 Thai *P. luminescens* isolates were distributed as groups or single isolates on 4 branches of the tree. Group 1 contained 45 Thai isolates and included sequences belonging to *P. luminescens* subsp. *hainanensis*, group 2 contained 8 Thai isolates and a reference sequence for *P. luminescens* subsp. *akhurstii*, group 3 contained 1 Thai isolate and no reference sequence, and group 4 contained two Thai isolates that were most closely related to *P. luminescens* subsp. *laumondii*. The *P. asymbiotica* isolate was on a distinct branch of the tree. Geographical mapping of the largest phylogenetic group containing the 45 isolates residing in group 1 demonstrated that these were recovered from numerous provinces (Table 2).

### Molecular characterization of EPNs

Nighty-five EPNs were identified using BLASTN searches of partial sequences of 28S rDNA and ITS, and the relationship described between EPNs and their bacterial symbionts. The remaining 31 EPNs were lost through fungal contamination. The EPNs associated with 62/69 *Xenorhabdus* isolates are shown in Figure 2. *X. stockiae* were associated with *S. websteri*, and *X. miraniensis* were associated with *S. khoisanae*. The EPNs associated with 33/57 *Photorhabdus* isolates are shown in Figure 3. The single isolate of *P. asymbiotica* was associated with *H. indica*. The remaining 32 isolates of *P. luminescens* were associated with *H. indica* (n = 26), *H*. sp. SGmg3 (n = 3), *H*. sp. SGgi (n = 1), *H. baujardi* (n = 1) and *H. bacteriophora* (n = 1).

## Discussion

The primary objective of this study was to isolate *Xenorhabdus* and *Photorhabdus* spp. from a geographical region that has been under-explored, and to phylogenetically characterize these isolates. Additional objectives were to determine the characteristics of the soil from which EPNs were isolated, characterize the EPN phylogeny, and describe the bacterial/EPN partners.

Phylogenetic analysis of 69 *Xenorhabdus* isolates demonstrated that *X. stockiae* predominated, with a small number of *X. miraniensis* (n = 3). Isolation of *X. stockiae* is consistent with the previous isolation from the environment in Thailand of *X. stockiae* strain TH01, the *recA* sequence of which fell within the largest phylogenetic cluster of 52 *X. stockiae* isolates identified here. A further 14 isolates were most closely related to *X. stockiae*, but showed evidence of evolutionary divergence. The *Steinernema* host species associated previously with *X. stockiae* isolated in Thailand was reported to be *S. siamkayai* [6], [7], and the relationship found is this study between *X. stockiae* and the host nematode *S. websteri* is a new observation. Elsewhere, *S. websteri* has been reported to be associated with *X. nematophila* [19]. *X. miraniensis* has been isolated previously from Australia but not from Thailand [7]. The three *X. miraniensis* isolates described in this study were from the same sub-district of Chaiyaphum and were associated with *S. khoisanae*. This nematode host has been reported previously in association with an unknown species of *Xenorhabdus* in South Africa [19].

Phylogenetic analysis of 57 *Photorhabdus* isolates demonstrated that 56 of these were *P. luminescens*, which could be sub-divided into several subspecies including subsp. *hainanensis* and subsp. *akhurstii*, together with isolates that were closely related to subsp. *laumondii*. *P. luminescens* subsp. *hainanensis* and subsp. *laumondii* have not been isolated previously from Thailand, but *P. luminescens* subsp. *akhurstii* was isolated previously from *H. indica* MP17 in Khon Kaen, from *H. indica* MP111 in Krabi and from a potentially new *Heterorhabditis* sp. MP68 in Kanchanaburi, Thailand [8]. *P. luminescens* subsp. *akhurstii* was restricted in our study to *H. indica*, while *P. luminescens* subsp. *hainanensis* was isolated from *H. indica, H.* sp. SGgi and *H.* sp. SGmg3. Elsewhere, subsp. *akhurstii* has been found in association with *H. bacteriophora* (in Iran, Hungary, Argentina and the USA) and *H. indica* (China) [8], and *P. luminescens* subsp. *hainanensis* has been isolated from an unknown *Heterorhabditis* sp. in China [6]. Isolates of *P. luminescens* of unknown subspecies were isolated in our study from *H. baujardi* and *H. bacteriophora*. *H. bacteriophora* has been associated previously with *P. luminescens* subsp. *akhurstii*, *caribbeanensis*, *kayaii*, *kleinii*, *laumondii* and *luminescens*, and *P. temperata* subsp. *cinerea*, *khanii*, *stackebrandtii* [8]. *H. baujardi* has been described in Vietnam [20], Brazil [21] and Cameroon [22], and in Brazil was found to carry *P. luminescens* [23].

A single isolate of *P. asymbiotica* was also cultured, which was in association with the nematode *H. indica*. It is likely that a more detailed characterization might assign this nematode to the *gerradi* subspecies that was shown to vector the Australian isolate, *P. asymbiotica* Kingscliff [24]. *P. asymbiotica* is an emerging pathogen that has been reported to cause locally invasive soft tissue infection and disseminated bacteremia. Clinical cases have been identified in both Australia and the USA [5], [24]. Our study represents the first reported isolation of *P. asymbiotica* from the Asian continent, and is an important indicator for the potential for clinical infection with this pathogen.

Our study demonstrated that EPNs could be isolated from diverse soil types in Thailand with a wide temperature, moisture and pH range. *Steinernema* or *Heterorhabditis* nematodes positive for *Xenorhabdus* and *Photorhabdus* spp. were isolated from 28% and 27% of the 159 sampling sites, respectively. Isolation of *Steinernema* has been reported from numerous countries across Europe, with rates of isolation varying from 2.2% to 36.8% [25]. *Heterorhabditis* is distributed throughout North and South America, Australia, Europe, Asia and Africa [6], [7]. In Thailand, the host nematode species isolated to date in association with *Xenorhabdus* and *Photorhabdus* have been limited to *S. siamkayai* (associated with *X. stockiae* [6], [7]) and *H. indica* (associated with *P. luminescens* [8]). Our isolation of the EPNs *S. websteri*, *S. khoisanae*, *H*. sp. SGmg3, *H*. sp. SGgi, *H. baujardi* and *H. bacteriophora* are new observations in Thailand.

The whole genome sequences of *Xenorhabdus* and *Photorhabdus* contain numerous genes encoding proteins and secondary metabolite synthetic enzymes that presumably make compounds that have a role in killing the insect and protection against other invading microorganisms in the infected insect cadaver [26]. The secondary metabolites have diverse chemical structures and a wide range of bioactive properties including antibiotic, antimycotic, insecticidal and nematicidal activity. For example, *X. nematophilia* produces xenocoumacins [26], and *Photorhabdus* all produce stilbene derivatives [27]. These genera also produce a range of bioactive protein molecules including the *Photorhabdus* proteins which have activity against other bacteria such as lumicins [28] or against the insect host, such as Mcf, Tc toxins and the PirAB toxins which show larvicidal activity against the vectors of dengue [29], [30]. We propose that the findings from this study could form a starting point for the rational choice of isolates for future studies focused on the discovery of novel antimicrobial and insecticidal compounds.
